# Kennedy’s Concentrated Extract of Pinus Canadensis

**Published:** 1871

**Authors:** J. Marion Sims


### *Kennedy's Concentrated Extract of Pinus Canadensis.*

By J. Marion Sims, M.D.

For many years Mr. Kennedy was engaged in making Hemlock Extract for tanner's use, which he shipped in large quantities to various parts of the country. The workmen engaged in manufacturing this impure commercial extract, accidently discovered that it was a valuable application in cuts and bruises, and in some cutaneous diseases, and also that it was a valuable remedy in diarrhœa and dysentery. One of his workmen. who had experienced the benefits of this crude article in a case of diarrhœa, concluded to try it locally for hæmorrhoids, a disease from which he had suffered for ten or twelve years. In five or six days he found himself greatly relieved, and in three months he was wholly cured.

All these facts fell under the personal observation of Mr. Kennedy, and he became so fully convinced of the value of the Hemlock Extract as a remedial agent that he determined to make an article medicinally pure, which he has for some time placed on the market. His method and care in making this new remedy will, I am sure, give us an article of uniform strength and purity. He extracts the virtues of the bark by pure distilled water; the temperature of which is never allowed to exceed 150 degrees F. The infusion thus made is then evaporated, *in vacuo*, from about twenty degrees (by what the tanners call the Barkometer) to 250 degrees, which makes a constant and uniform Fluid Extract, without the addition of acid or alcohol, and which does not ferment in any climate or under any extreme of temperature.

I have had but a limited experience with this new Extract of Pinus Canadensis, but I am so well satisfied of its value that I am anxious to call the attention of the profession to it. I have used it for about eight months in some affections of the rectum, vagina and cervix uteri; I have used it, considerably diluted, as a vaginal wash, with great success; but I prefer to apply it to the os tincæ on cotton wool, either pure or mixed with glycerine or glycerine and rose-water. Thus applied, it should remain intact for two or three, or even four days, and then be renewed. In this way I have seen chronic granular vaginitis remedied in a few days, that had resisted the ordinary remedies for weeks; and I have seen granula erosions, with leucorrhœa, disappear very rapidly under its use. I have not time to do more than call the attention of my professional brethren to this new extract, which I am sure will soon be recognized as a valuable addition to our Materia Medica.—*Medical Gazette.*

